# Efficacy and safety of esketamine nasal spray by sex in patients with treatment-resistant depression: findings from short-term randomized, controlled trials

**DOI:** 10.1007/s00737-021-01185-6

**Published:** 2022-01-01

**Authors:** Robyn R. Jones, Marlene P. Freeman, Susan G. Kornstein, Kimberly Cooper, Ella J. Daly, Carla M. Canuso, Susan Nicholson

**Affiliations:** 1 Office of the Chief Medical Officer, Health of Women, Johnson & Johnson, 410 George Street, New Brunswick, NJ 08901 USA; 2 Department of Psychiatry, Harvard Medical School, Boston, MA USA; 3 Departments of Psychiatry and Obstetrics & Gynecology, Virginia Commonwealth University School of Medicine, Richmond, VA USA; 4 Department of Statistics & Decision Sciences, Janssen Research & Development, LLC, Spring House, PA USA; 5 Department of Neuroscience Medical Affairs, Janssen Scientific Affairs, LLC, Titusville, NJ USA; 6 Department of Neuroscience Clinical Development, Janssen Research & Development, LLC, Titusville, NJ USA

**Keywords:** Esketamine, s-Ketamine, Treatment-resistant depression, Sex

## Abstract

The objective of this analysis was to determine if there are sex differences with esketamine for treatment-resistant depression (TRD). Post hoc analyses of three randomized, controlled studies of esketamine in patients with TRD (TRANSFORM-1, TRANSFORM-2 [18–64 years], TRANSFORM-3 [≥ 65 years]) were performed. In each 4-week study, adults with TRD were randomized to esketamine or placebo nasal spray, each with a newly initiated oral antidepressant. Change from baseline to day 28 in Montgomery-Åsberg Depression Rating Scale (MADRS) total score was assessed by sex in pooled data from TRANSFORM-1/TRANSFORM-2 and separately in data from TRANSFORM-3 using a mixed-effects model for repeated measures. Use of hormonal therapy was assessed in all women, and menopausal status was assessed in women in TRANSFORM-1/TRANSFORM-2. Altogether, 702 adults (464 women) received ≥ 1 dose of intranasal study drug and antidepressant. Mean MADRS total score (SD) decreased from baseline to day 28, more so among patients treated with esketamine/antidepressant vs. antidepressant/placebo in both women and men: TRANSFORM-1/TRANSFORM-2 women—esketamine/antidepressant -20.3 (13.19) vs. antidepressant/placebo -15.8 (14.67), men—esketamine/antidepressant -18.3 (14.08) vs. antidepressant/placebo -16.0 (14.30); TRANSFORM-3 women—esketamine/antidepressant -9.9 (13.34) vs. antidepressant/placebo -6.9 (9.65), men—esketamine/antidepressant -10.3 (11.96) vs. antidepressant/placebo -5.5 (7.64). There was no significant sex effect or treatment-by-sex interaction (*p* > 0.35). The most common adverse events in esketamine-treated patients were nausea, dissociation, dizziness, and vertigo, each reported at a rate higher in women than men. The analyses support antidepressant efficacy and overall safety of esketamine nasal spray are similar between women and men with TRD. The TRANSFORM studies are registered at clinicaltrials.gov (identifiers: NCT02417064 (first posted 15 April 2015; last updated 4 May 2020), NCT02418585 (first posted 16 April 2015; last updated 2 June 2020), and NCT02422186 (first posted 21 April 2015; last updated 29 September 2021)).

## Introduction

Women compared to men exhibit a twofold higher risk of major depressive disorder (MDD) (Hasin et al. 2005), and differences in clinical presentation and comorbidities. In the Sequenced Treatment Alternatives to Relieve Depression (STAR*D) study of outpatients with nonpsychotic MDD, women had approximately 2 years earlier onset of their first major episode, greater severity of MDD, and approximately 5.6 months longer duration of depressive episodes, compared to the men (Marcus et al. 2005). Women were more likely to experience concurrent symptoms consistent with generalized anxiety disorder, somatoform disorder, bulimia, and atypical depression, and less likely to have concurrent obsessive compulsive, alcohol abuse, and drug abuse disorders (Marcus et al. 2005).

Likewise, response to antidepressants can vary between women and men, including differences in time to response, response rate, and adverse effects (Kornstein et al. 2000; Khan et al. 2005; Berlanga and Flores-Ramos 2006; Young et al. 2009; Sramek et al. 2016). Various factors have been suggested to explain disparity in antidepressant response between women and men, including differences in neuronal circuitry, hormone levels, and drug metabolism (Bigos et al. 2009; Dalla et al. 2010; Fernández-Guasti et al. 2012). For example, menopausal status and sex hormone therapy impacted treatment responses among women receiving selective serotonin reuptake inhibitor (SSRI) or a serotonin and norepinephrine reuptake inhibitor (SNRI) therapy in some studies (Kornstein et al. 2000; Grigoriadis et al. 2003; Thase et al. 2005; Pinto-Meza et al. 2006), but not in others (Quitkin et al. 2002; Cassano et al. 2005; Kornstein et al. 2013, 2014). A more recent study found no difference in efficacy for women compared to men with intravenous ketamine given as an acute treatment in patients with treatment-resistant depression (TRD) (Freeman et al. 2019). These mixed findings underscore the value of investigating if there are sex differences in response to newly approved antidepressants.

Esketamine (the S-enantiomer of ketamine racemate), a first-in-class glutamatergic N-methyl-D-aspartate (NMDA) receptor antagonist, has been approved by the US Food and Drug Administration, the European Medicines Agency, and multiple other health authorities for TRD, in conjunction with an oral antidepressant (Spravato Prescribing Information 2020; Spravato Summary of Product Characteristics 2020). The approval of esketamine nasal spray was based, in large part, on efficacy and safety findings from phase 2 and phase 3 studies in patients with TRD (Daly et al. 2018, 2019; Popova et al. 2019; Fedgchin et al. 2019; Wajs et al. 2020; Ochs-Ross et al. 2020). The database from these clinical development trials forms the basis for additional investigations that may facilitate clinical management of patients treated with esketamine nasal spray in real world clinical practice.

A post hoc analysis of data from three short-term phase 3 studies (Popova et al. 2019; Fedgchin et al. 2019; Ochs-Ross et al. 2020) was conducted to assess the effect of sex on the efficacy and safety of esketamine nasal spray in patients with TRD. The primary aims of these analyses are to determine whether there are differences between women and men based on improvement of depressive symptoms, comorbid anxiety, and response and remission rates with esketamine. Secondarily, among women, the aims were to determine if the aforementioned outcomes are affected by menopausal status or by use of hormonal therapy and if there are clinical factors that differentiated responders from non-responders, supporting data-informed decision-making for women.

## Materials and methods

The methods of the TRANSFORM studies are published elsewhere (Popova et al. 2019; Fedgchin et al. 2019; Ochs-Ross et al. 2020). Study methods salient to the work reported here are summarized below.

### Ethical practices

Institutional review boards/ethics committees approved the study protocols and their amendments, written consent was obtained from all patients before study participation, and the studies are registered at clinicaltrials.gov (identifiers: NCT02417064, NCT02418585, and NCT02422186).

### Study design

The TRANSFORM trials were phase 3, double-blind, active-controlled, multicenter studies of esketamine nasal spray in patients with TRD. The trials comprised three phases: (1) 4-week screening/prospective observational phase assessing treatment response to the current antidepressant(s), (2) 4-week double-blind treatment phase with esketamine or placebo nasal spray combined with a newly initiated oral antidepressant, and (3) post-treatment follow-up phase assessing safety (TRANSFORM-1 and TRANSFORM-2: up to 24 weeks; TRANSFORM-3: 2 weeks). The studies were conducted between August 2015 and February 2018.

### Patients

The studies enrolled outpatients (TRANSFORM-1 and TRANSFORM-2: aged 18–64 years; TRANSFORM-3: ≥ 65 years) with recurrent, moderate-to-severe MDD (DSM-5 diagnosis [APA 2013] without psychotic features, confirmed by the Mini International Neuropsychiatric Inventory (MINI) [Sheehan et al. 1998]). At randomization, participants had TRD, defined as non-response to two or more oral antidepressants, taken at an adequate dosage and for an adequate duration, during the current episode.

Key exclusion criteria included suicidal ideation with intent to act within the prior 6 months or suicidal behavior within the prior year; diagnosis of psychotic or bipolar disorders; recent history (within prior 6 months) of moderate or severe substance use disorder; and positive test result(s) for specified drugs of abuse. Full lists of the inclusion and exclusion criteria for each study are published (Popova et al. 2019; Fedgchin et al. 2019; Ochs-Ross et al. 2020).

### Study drug dosing

Patients continued taking their current antidepressant during the 4-week screening/prospective observational phase. At the end of the screening phase, non-responders (≤ 25% improvement in Montgomery-Åsberg Depression Rating Scale [MADRS] total score from week 1 to week 4) discontinued all current antidepressant treatment(s) and were randomized to double-blind treatment, consisting of twice-weekly esketamine nasal spray or matching (appearance, taste, and packaging) placebo nasal spray, each combined with a newly initiated oral antidepressant (SSRI or SNRI) taken daily. The doses of esketamine were 56 mg (starting dose) and 84 mg in the TRANSFORM-1 (fixed dose) and TRANSFORM-2 (flexible dose) studies and 28 mg (starting dose and a dose option during the study), 56 mg, and 84 mg in TRANSFORM-3 (flexible dose).

### Assessments

Improvement in symptoms of depression was assessed by the MADRS (Williams and Kobak 2008), which was administered by independent, blinded raters at baseline and subsequent visits during the double-blind treatment phase. In addition, patient-reported outcomes included an assessment of function using the Sheehan Disability Scale (SDS) (Leon et al. 1997), depressive symptoms using the Patient Health Questionnaire 9-item (PHQ-9) (Spitzer et al. 1999), and severity of anxiety using the Generalized Anxiety Disorder 7-item (GAD-7) Scale (GAD-7 in TRANSFORM-1 and TRANSFORM-2 only) (Spitzer et al. 2006).

The clinician-rated Massachusetts General Hospital Female Reproductive Lifecycle and Hormones Questionnaire (Freeman et al. 2013), Module 1, was used to assess and prospectively document reproductive lifecycle status (pre-menopausal, peri-menopausal, or post-menopausal), history of worsening mood during the luteal phase of the menstrual cycle, length and regularity of menstrual cycles, and use of exogenous hormones, including hormonal oral contraceptives (OC) and hormone replacement therapy (HRT). In reporting menopause status, the investigator selected the appropriate choice from the following: pre-menopausal; peri-menopausal (irregular periods and/or other symptoms of peri-menopause, such as hot flashes not explained by other reasons); post-menopausal non-surgical (> 12 months of amenorrhea); and post-menopausal surgical (status post bilateral oophorectomy).

Treatment-emergent adverse events were assessed throughout the study.

### Statistical analyses

Efficacy was analyzed in a data set that included all randomized patients who received at least 1 dose of intranasal study drug (esketamine or placebo) and 1 dose of oral antidepressant, and adverse events were analyzed in a data set that included all patients that received at least 1 dose of either medication. Data from the TRANSFORM-1 and TRANSFORM-2 studies were pooled.

Baseline characteristics and psychiatric history were summarized by sex using descriptive statistics. The prevalence of comorbid anxiety at baseline was determined using one of the following: generalized anxiety disorder current, panic disorder current, social anxiety disorder current, post-traumatic stress disorder current, or obsessive–compulsive disorder current based on the MINI, or having screening and baseline GAD-7 total score ≥ 10 (for TRANSFORM-1/TRANSFORM-2 only).

The primary efficacy endpoint in the TRANSFORM studies—change from baseline to endpoint (day 28) in MADRS total score—was analyzed by sex using a mixed-effects model for repeated measures (MMRM). The model included baseline MADRS total score as a covariate, and treatment, study (TRANSFORM-1/TRANSFORM-2 only), region, oral antidepressant class (SNRI or SSRI), day, sex, day-by-treatment, treatment-by-sex, and day-by-treatment-by-sex interaction as fixed effects, and a random patient effect. Changes in SDS and PHQ-9 were analyzed using the MMRM model described for the primary efficacy endpoint, but using the respective baseline score (SDS, PHQ-9) as covariate. Change in GAD-7 was analyzed using analysis of covariance with baseline GAD-7 as a covariate and treatment, region, oral antidepressant class, sex, and treatment-by-sex as factors.

Response rate (defined as ≥ 50% decrease from baseline MADRS total score) and remission rate (defined as MADRS ≤ 12) at day 28 were analyzed by treatment group and sex using the generalized Cochran-Mantel–Haenszel (CMH) test. Response rate was also evaluated by menopausal status and by use of sex hormones ([i.e., HRT or OCs], yes or no) among women. In other analyses, frequency distributions were provided by treatment group and sex for adverse events as a measure of safety.

## Results

Across the TRANSFORM trials, 711 patients were randomized to treatment, 6 of whom did not receive intranasal study drug and 3 additional patients did not receive either study drug. Thus, efficacy and safety were evaluated in 702 patients (464 [66.1%] women and 238 [33.9%] men) and 705 patients, respectively. Most randomized patients (women: 427/471, 90.7%; men: 207/240, 86.3%) completed double-blind treatment.

     Within each of the TRANSFORM studies, women and men were similar, in general, with respect to demographic and baseline clinical characteristics (Table 1). Per protocol, patients in TRANSFORM-3 were older (≥ 65 years). Approximately half of women in the TRANSFORM-1 and TRANSFORM-2 studies reported being pre-menopausal (182/379, 48.0%). Of note, in the TRANSFORM-1/2 studies, the mean age at MDD diagnosis (32.8 and 31.2 years old, respectively) and mean duration of the current episode (161.5 and 181.4 weeks, respectively) were similar between women and men. In TRANSFORM-3, women received an MDD diagnosis at an earlier age (41.6 vs. 45.6 years old for men) and had a shorter current episode of MDD (188.6 vs. 260.3 weeks for men).

**Table 1:** Demographic and baseline characteristics by sex in short-term randomized, controlled TRD trials

	TRANSFORM-1/TRANSFORM-2			TRANSFORM-3		
Characteristic	Women *N* = 379	Men *N* = 186	Total *N* = 565	Women *N* = 85	Men *N* = 52	Total *N* = 137
Age, years						
Mean (SD)	46.6 (11.05)	44.9 (12.22)	46.1 (11.46)	70.3 (4.9)	69.5 (3.8)	70.0 (4.52)
Range	(18; 64)	(18; 64)	(18; 64)	(65; 86)	(65; 79)	(65; 86)
Race, *n* (%)						
American Indian or Alaskan Native	1 (0.3)	0	1 (0.2)	0	0	0
Asian	5 (1.3) (1.2)	2 (1.1)	7 (1.2)	0	0	0
Black or African American	23 (6.1)	7 (3.8)	30 (5.3)	0	0	0
White	302 (79.7)	168 (90.3)	470 (83.2)	81 (95.3)	49 (94.2)	130 (94.9)
Other	25 (6.6)	4 (2.2)	29 (5.1)	0	0	0
Multiple	1 (0.3)	2 (1.1)	3 (0.5)	2 (2.4)	2 (3.8)	4 (2.9)
Not reported	22 (5.8)	3 (1.6)	25 (4.4)	1 (1.2)	1 (1.9)	2 (1.5)
Unknown	0	0	0	1 (1.2)	0	1 (0.7)
Body mass index (kg/m^2^)						
Mean (SD)	28.4 (6.60)	28.7 (5.59)	28.5 (6.28)	28.9 (6.3)	28.9 (4.5)	28.9 (5.64)
Range	(17; 56)	(16; 56)	(16; 56)	(16; 45)	(22; 42)	(16; 45)
Menopause status^a^, *n* (%)						
Pre-menopausal	182 (48.0)	NA		NA	NA	
Peri-menopausal	24 (6.3)	NA		NA	NA	
Post-menopausal—non-surgical	120 (31.7)	NA		NA	NA	
Post-menopausal—surgical	53 (14.0)	NA		NA	NA	
Regular menstrual cycles, *n* (%)	N = 203					
Yes	160^a^ (78.8)	NA		NA	NA	
No	43 (21.2)	NA		NA	NA	
Median length of typical menstrual cycle (days)	28.0	NA		NA	NA	
History of worsening luteal phase, *n* (%)	N = 203					
Yes	43 (21.2)	NA		NA	NA	
No	160^a^ (78.8)	NA		NA	NA	
Employment status^b^, *n* (%)						
Any type of employment	218 (57.5)	107 (57.5)	325 (57.5)	14 (16.5)	10 (19.2)	24 (17.5)
Any type of unemployment	122 (32.2)	65 (34.9)	187 (33.1)	4 (4.7)	4 (7.7)	8 (5.8)
Other	39 (10.3)	14 (7.5)	53 (9.4)	67 (78.8)	38 (73.1)	105 (76.6)
Region, *n* (%)						
Europe	1542 (37.5)	77 (41.4)	219 (38.8)	40 (47.1)	19 (36.5)	59 (43.1)
North America	151 (39.8)	93 (50.0)	244 (43.2)	40 (47.1)	30 (57.7)	70 (51.1)
Age when diagnosed with MDD, years						
Mean (SD)	32.8 (12.69)	31.2 (132.69)	32.3 (12.70)	41.6 (15.9)	45.6 (16.5)	43.1 (16.2)
Range	(9; 61)	(5; 64)	(5; 64)	(10; 75)	(11; 77)	(10; 77)
Duration of current episode, weeks						
Mean (SD)	161.5 (236.4)	181.4 (276.5)	168.1 (250.2)	188.6 (279.5)	260.3 (423.6)	215.8 (341.7)
Range	(6; 2288)	(12; 2028)	(6; 2288)	(8; 1700)	(8; 2184)	(8; 2184)
No. of previous antidepressants^c,d^, *n* (%)						
1 or 2	245 (65.0)	110 (59.2)	355(63.1)	54 (63.5)	30 (57.7)	84 (61.3)
≥ 3	132 (35.0)	76 (40.8)	208 (36.9)	31 (36.4)	22 (42.3)	53 (38.7)
Class of oral antidepressant^e^, *n* (%)						
SNRI	236 (63.3)	112 (60.2)	348 (61.6)	34 (40.0)	27 (51.9)	61 (44.5)
SSRI	143 (37.7)	74 (39.8)	217 (38.4)	51 (60.0)	25 (48.1)	76 (55.5)
Oral antidepressant, *n* (%)						
Duloxetine	174 (45.9)	83 (44.6)	257 (45.5)	25 (29.4)	23 (44.2)	48 (35.0)
Escitalopram	74 (19.5)	37 (19.9)	111 (19.6)	36 (42.4)	14 (26.9)	50 (36.5)
Sertraline	68 (17.9)	37 (19.9)	105 (18.6)	14 (16.5)	11 (21.2)	25 (18.2)
Venlafaxine XR	63 (16.6)	29 (15.6)	92 (16.3)	10 (11.8)	4 (7.7)	14 (10.2)
CGI-S						
Mean (SD)	5.1 (0.67)	5.1 (0.72)	5.1 (0.68)	5.0 (0.75)	5.0 (0.85)	5.0 (0.79)
MADRS total score						
Mean (SD)	37.7 (5.73)	36.8 (5.21)	37.4 (5.57)	35.2 (6.41)	35.2 (5.78)	35.2 (6.16)
PHQ-9 total score						
Mean (SD)	20.6 (3.67)	20.3 (3.91)	20.5 (3.75)	17.6 (5.53)	17.4 (5.87)	17.5 (5.65)
SDS total score						
Mean (SD)	24.5 (4.09)	23.9 (4.40)	24.3 (4.20)	23.2 (5.12)	21.3 (5.49)	22.3 (5.36)
GAD-7 total score						
Mean (SD)	13.4 (5.23)	12.9 (5.02)	13.2 (5.16)	NA	NA	NA

Abbreviations: *CGI-S* Clinical Global Impression–Severity; *MDD* major depressive disorder; *NA* not applicable or not available, not administered; *PHQ* Patient Health Questionnaire; *SNRI* serotonin and norepinephrine reuptake inhibitor; *SSRI* selective serotonin reuptake inhibitor; *TRD* treatment-resistant depression

^a^Data from the Massachusetts General Hospital Female Reproductive Lifecycle and Hormones Questionnaire, Module I (Freeman et al. 2013), in the TRANSFORM-1 and TRANSFORM-2 studies

^b^Any type of employment includes any category containing “employed,” sheltered work, housewife or dependent husband, and student; any type of unemployment includes any category containing “unemployed”; other includes retired and no information available

^c^In accordance with the trial protocols, patients entering the induction phase had non-response to at least 2 oral antidepressant medications prior to randomization. The data presented is the number of antidepressant medications with non-response (defined as ≤ 25% improvement) taken for at least 6 weeks during the current episode

^d^Ns for the previous antidepressant medications in TRANSFORM-1/TRANSFORM-2 are 377, 186, and 563 for women, men, and total patients, respectively

^e^Assigned by the investigator at randomization

The majority (71.5%) of patients in the TRANSFORM-1/2 studies had comorbid anxiety symptoms at baseline, with no difference in prevalence between sexes (Table 2). Among the common medical comorbidities reported were hypertension, cardiovascular disease, diabetes, and thyroid disease. The prevalence of hypertension was balanced between sexes, whereas cardiovascular disease and diabetes were more common in older men than older women, and thyroid disease was more common in both older and younger women than men. Of note, when we refer to younger women, here and elsewhere, we are referring to those enrolled in the TRANSFORM-1/2 studies (who were 18–64 years) and older women were those enrolled in TRANSFORM-3 (who were 65 years and older). Usage of concomitant medications, which was in line with these comorbidities, was generally balanced between treatment groups among women and men, with the exception of higher levothyroxine usage by women (Table 3). In the TRANSFORM-1/2 studies, hormonal therapy (including HRT and OCs) was taken by 34.8% and 21.4% of pre-menopausal women in the esketamine/antidepressant and antidepressant/placebo groups respectively, 40% and 22.2% of peri-menopausal women, and 3.7% and 6.2% of post-menopausal women in the respective treatment groups. Hormonal therapy use by women in TRANSFORM-3 was uncommon (Table 3).

**Table 2:** Comorbidities of study patients in short-term randomized, controlled TRD trials

	Number (%) of patients					
	TRANSFORM-1/TRANSFORM-2			TRANSFORM-3		
Comorbidities	Women *N* = 379	Men *N* = 186	Total *N* = 565	Women *N* = 85	Men *N* = 52	Total *N* = 137
Anxiety^a^	272 (71.8%)	132 (71.0%)	404 (71.5%)	NA	NA	NA
Incidental surgery	166 (43.8%)	55 (29.6%)	221 (39.1%)	37 (43.5%)	19 (36.5%)	56 (40.9%)
Hypertension	79 (20.8%)	38 (20.4%)	117 (20.7%)	46 (54.1%)	28 (53.8%)	74 (54.0%)
Allergies	69 (18.2%)	31 (16.7%)	100 (17.7%)	11 (12.9%)	4 (7.7%)	15 (10.9%)
Thyroid disease	57 (15.0%)	10 (5.4%)	67 (11.9%)	23 (27.1%)	6 (11.5%)	29 (21.2%)
GERD	48 (12.7%)	15 (8.1%)	63 (11.2%)	13 (15.3%)	8 (15.4%)	21 (15.3%)
Trauma	27 (7.1%)	31 (16.7%)	58 (10.3%)	5 (5.9%)	3 (5.8%)	8 (5.8%)
Cardiovascular disease	24 (6.3%)	13 (7.0%)	37 (6.5%)	10 (11.8%)	12 (23.1%)	22 (16.1%)
Diabetes	25 (6.6%)	8 (4.3%)	33 (5.8%)	13 (15.3%)	13 (25.0%)	26 (19.0%)
Oncology	13 (3.4%)	5 (2.7%)	18 (3.2%)	16 (18.8%)	11 (21.2%)	27 (19.7%)
Skin disorder	23 (6.1%)	13 (7.0%)	36 (6.4%)	3 (3.5%)	4 (7.7%)	7 (5.1%)
Infection	25 (6.6%)	6 (3.2%)	31 (5.5%)	2 (2.4%)	2 (3.8%)	4 (2.9%)
Respiratory disease	12 (3.2%)	2 (1.1%)	14 (2.5%)	1 (1.2%)	0	1 (0.7%)
Parathyroid disease	2 (0.5%)	1 (0.5%)	3 (0.5%)	0	1 (1.9%)	1 (0.7%)

Data presented in descending order of frequency for all patients

*GERD* gastroesophageal reflux disease; *NA* not available; *TRD* treatment-resistant depression

^a^Comorbid anxiety defined by one of the following at screening: generalized anxiety disorder current, panic disorder current, social anxiety disorder current, post-traumatic stress disorder current, or obsessive–compulsive disorder current by MINI, or having GAD-7 total score ≥ 10 at screening and at baseline. GAD-7 was not conducted in the TRANSFORM-3 study

**Table 3:** Concomitant medications most frequently used during double-blind treatment in short-term randomized, controlled TRD trials

	Women		Men	
Specific or category of concomitant medication	Esketamine + antidepressant *N* = 282	Antidepressant + placebo *N* = 184	Esketamine + antidepressant *N* = 136	Antidepressant + placebo *N* = 103
Benzodiazepine	140 (49.6%)	86 (46.7%)	66 (48.5%)	36 (35.0%)
Analgesic	79 (28.0%)	57 (31.0%)	34 (25.0%)	24 (23.3%)
Antihypertensive	62 (22.0%)	53 (28.8%)	33 (24.3%)	32 (31.1%)
Lipid-lowering agent	45 (16.0%)	36 (19.6%)	32 (23.5%)	28 (27.2%)
Proton pump inhibitor	40 (14.2%)	28 (15.2%)	24 (17.6%)	11 (10.7%)
Beta-blocker	34 (12.1%)	21 (11.4%)	21 (15.4%)	9 (8.7%)
Thyroid medications	37 (13.1%)	35 (19.0%)	5 (3.7%)	8 (7.8%)
Levothyroxine	36 (12.8%)	33 (17.9%)	5 (3.7%)	7 (6.8%)
Hormonal therapy^a^	49 (17.4%)	24 (13.0%)	NA	NA
TRANSFORM-1/2				
Pre-menopausal	39/112 (34.8%)	15/70 (21.4%)		
Peri-menopausal	6/15 (40.0%)	2/9 (22.2%)		
Post-menopausal	4/108 (3.7%)	4/65 (6.2%)		
TRANSFORM-3	0/45 (0.0%)	3/40 (7.5%)		

The table lists, in descending order of frequency for all patients, all specific or categories of concomitant medication with a usage rate during double-blind treatment of ≥ 10% in either treatment group, without regard to sex

^a^Includes hormone replacement therapy and oral contraceptives

Mean MADRS total score decreased from baseline to day 28, with greater improvement among those treated with esketamine/antidepressant compared to antidepressant/placebo among both women and men. The mean MADRS change (SD) at day 28 for the esketamine/antidepressant and antidepressant/placebo groups were -20.3 (13.19) vs. -15.8 (14.67), respectively, among the women and -18.3 (14.08) vs. -16.0 (14.30), respectively, among the men in TRANSFORM-1/TRANSFORM-2; and -9.9 (13.34) vs. -6.9 (9.65), respectively, among the women and -10.3 (11.96) vs. -5.5 (7.64), respectively, among the men in TRANSFORM-3 (Table 4). The analysis failed to show any significant sex effect or treatment-by-sex interaction (*p* > 0.35).

**Table 4:** MADRS total score: change from baseline to day 28 of double-blind phase of short-term randomized, controlled TRD trials by sex and treatment group

	TRANSFORM-1/TRANSFORM-2				TRANSFORM-3			
	Women		Men		Women		Men	
	Esketamine + antidepressant	Antidepressant + placebo	Esketamine + antidepressant	Antidepressant + placebo	Esketamine + antidepressant	Antidepressant + placebo	Esketamine + antidepressant	Antidepressant + placebo
Baseline								
* N*	235	144	108	78	45	40	27	25
Mean (SD)	37.7 (5.49)	37.7 (6.11)	36.9 (5.02)	36.7 (5.50)	35.7 (5.90)	34.5 (6.97)	35.2 (6.04)	35.1 (5.60)
Change to day 28								
* N*	215	138	95	70	39	36	24	24
Mean (SD)	-20.3 (13.19)	-15.8 (14.67)	-18.3 (14.08)	-16.0 (14.30)	-9.9 (13.34)	-6.9 (9.65)	-10.3 (11.96)	-5.5 (7.64)
MMRM analysis^a^								
Diff. of LS means^b^ (SE)	-4.5 (1.41)		-1.6 (2.04)		-3.4 (2.41)		-5.0 (3.05)	
95% CI on difference	-7.26, − 1.70		-5.60, 2.41		-8.14, 1.41		-11.05, 1.03	

MADRS total score ranges from 0 to 60; a higher score indicates a more severe condition. Negative change in score indicates improvement. Negative difference favors esketamine. The *p*-values were 0.6574 and 0.3993 for sex, and 0.3546 and 0.4937 for treatment-by-sex interaction in the TRANSFORM-1/2 and TRANSFORM-3 studies, respectively

*CI* confidence interval; *LS* least squares; *MADRS* Montgomery-Asberg Depression Rating Scale; *TRD* treatment-resistant depression

^a^Mixed model for repeated measures (MMRM) analysis with change from baseline as the response variable and the fixed effect model terms for study number (pooled only), treatment (esketamine + antidepressant, antidepressant + placebo) day, region, class of antidepressant (SNRI or SSRI), sex, and treatment-by-day, treatment-by-sex, and treatment-by-day-by-sex, and baseline value as a covariate

^b^Esketamine + antidepressant minus antidepressant + placebo

**Fig. 1:**
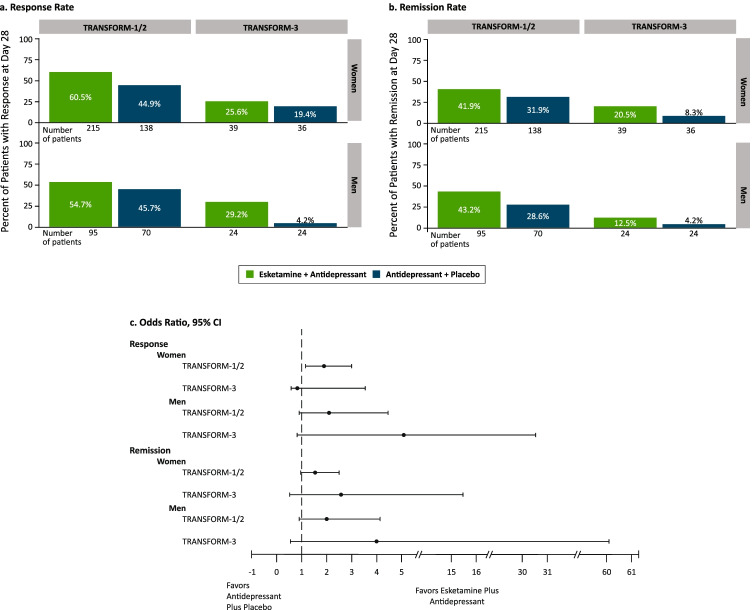
Response and remission rates by sex and treatment group in the TRANSFORM trials. *CI* = confidence interval. Notes: Response defined as ≥ 50% decrease from baseline Montgomery-Asberg Depression Rating Scale (MADRS) total score. Remission defined as MADRS total score ≤ 12. Odds ratio = odds of achieving response on esketamine + antidepressant divided by the odds of achieving response on antidepressant + placebo

**Fig. 2:**
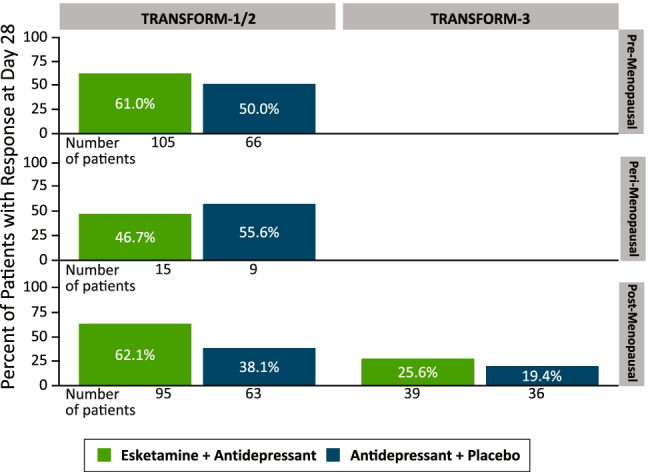
Response rates by menopausal status and treatment group in TRANSFORM trials. Notes: Response defined as ≥ 50% decrease from baseline Montgomery-Asberg Depression Rating Scale (MADRS) total score

In the TRANSFORM trials, the proportions of patients who were responders at day 28 and the proportion of patients in remission at day 28 were numerically higher among both women and men treated with esketamine/antidepressant as compared to antidepressant/placebo (Fig. 1). In TRANSFORM-3, the remission rate, but not response rate, was numerically higher among the older women compared to their male counterparts. In TRANSFORM-1/TRANSFORM-2, pre-menopausal and post-menopausal women treated with esketamine achieved similar response rates, and the same between treatment group trend was observed for response rate among the pre-menopausal and post-menopausal women (Fig. 2). In the cohort of peri-menopausal women (*n* = 25), response rate with esketamine/antidepressant was numerically lower than among pre-menopausal and post-menopausal women. In the TRANSFORM-1/2 studies, use of hormonal therapy (HRT or OCs) had an impact on response rate at day 28 in the antidepressant/placebo arm (hormone users: 73.7% [14/19]; hormone non-users: 40.3% [48/119]), but not in the esketamine/antidepressant arm (hormone users: 54.2% [28/48]; hormone non-users: 62.3% [104/167]).

**Fig. 3:**
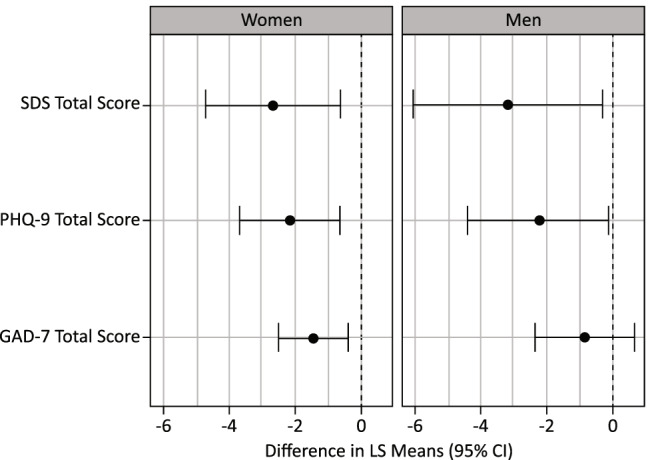
Difference in least square means for SDS, PHQ-9, and GAD-7 total score by sex in pooled TRANSFORM-1/TRANSFORM-2 trials. *CI* = confidence interval; *GAD-7* = Generalized Anxiety Disorder 7-item; *LS* = least squares; *PHQ-9* = Patient Health Questionnaire 9-item; *SDS* = Sheehan Disability Scale. Notes: SDS total score ranges from 0 to 30; a higher score indicates greater impairment. PHQ-9 total score ranges from 0 to 27; a higher score indicates greater depression. GAD-7 total score ranges from 0 to 21; a higher score indicates more anxiety. Negative change in SDS total score, PHQ-9 total score, and GAD-7 total score indicates improvement for each, and a negative difference favors esketamine

Treatment benefit of esketamine was also observed in terms of functioning and self-reported depression for both women and men in the pooled TRANSFORM-1/TRANSFORM-2 trials (Table 5, Fig. 3). The analysis for SDS and PHQ-9 failed to show any significant sex effect or treatment-by sex-interaction (*p* > 0.20). For GAD-7, there was no treatment-by-sex interaction (*p* > 0.52); however, the sex effect trended towards significance (*p* = 0.07; i.e., women showed greater change from baseline than men, regardless of treatment).

**Table 5:** Mean (SD) change from baseline to day 28 for SDS, PHQ-9, and GAD-7 total score by sex in pooled TRANSFORM-1/TRANSFORM-2 trials

	TRANSFORM-1/TRANSFORM-2			
	Women		Men	
	Esketamine + antidepressant	Antidepressant + placebo	Esketamine + antidepressant	Antidepressant + placebo
SDS total score				
* N*	177	115	84	60
Mean (SD)	-12.5 (9.30)	-9.6 (9.50)	-10.6 (9.22)	-7.4 (8.12)
PHQ-9 total score				
* N*	218	138	95	70
Mean (SD)	-12.3 (7.39)	-10.1 (7.99)	-10.9 (7.62)	-8.6 (8.25)
GAD-7 total score				
* N*	227	139	103	74
Mean (SD)	-8.1 (5.90)	-6.9 (5.78)	-6.7 (5.84)	-5.4 (5.98)

SDS total score ranges from 0 to 30; a higher score indicates greater impairment. PHQ-9 total score ranges from 0 to 27; a higher score indicates greater depression. GAD-7 total score ranges from 0 to 21; a higher score indicates more anxiety. Negative change in SDS total score, PHQ-9 total score, and GAD-7 total score indicates improvement for each

*GAD-7* Generalized Anxiety Disorder 7-item; *LS* least squares; *PHQ-9* Patient Health Questionnaire 9-item; *SD* standard deviation; *SDS* Sheehan Disability Scale

The most common adverse events (incidence > 20%) reported for esketamine/antidepressant were nausea, dissociation, dizziness, and vertigo (Table 6). Among esketamine-treated patients, the incidences of nausea and dissociation were higher among women than among men, regardless of age. The incidences of vertigo and dizziness were numerically higher and lower, respectively, among the younger women vs. younger men in the TRANSFORM-1/TRANSFORM-2 studies; the opposite by-sex trend for incidences of these events was observed among the older patients in TRANSFORM-3. While reported at a lower incidence overall (i.e., 9.3%), the incidence of increased blood pressure was numerically higher among younger men (12.8%) and older women (17.8%), than their counterparts. Overall, most adverse events occurred on nasal spray dosing days, were mild or moderate in severity, resolved the same day, and were generally not treatment limiting. With regard to dissociation events, their median duration ranged from 0.7 to 1 h across dosing sessions (within the post-dose observation period), a minority (3.1%) of events were classified as severe, and none were considered serious.

**Table 6:** Most frequently reported treatment-emergent adverse events in the double-blind treatment phase of short-term randomized, controlled TRD trials by sex and treatment group

	TRANSFORM-1/TRANSFORM-2				TRANSFORM-3			
	Women		Men		Women		Men	
	Esketamine + antidepressant *N* = 237	Antidepressant + placebo *N* = 144	Esketamine + antidepressant *N* = 109	Antidepressant + placebo *N* = 78	Esketamine + antidepressant *N* = 45	Antidepressant + placebo *N* = 40	Esketamine + antidepressant *N* = 27	Antidepressant + placebo *N* = 25
Total with AEs	209 (88.2%)	96 (66.7%)	92 (84.4%)	47 (60.3%)	34 (75.6%)	23 (57.5%)	17 (63.0%)	16 (64.0%)
Nausea	72 (30.4%)	11 (7.6%)	26 (23.9%)	8 (10.3%)	9 (20.0%)	3 (7.5%)	4 (14.8%)	0
Headache	52 (21.9%)	21 (14.6%)	18 (16.5%)	17 (21.8%)	6 (13.3%)	1 (2.5%)	3 (11.1%)	1 (4.0%)
Dizziness	53 (22.4%)	10 (6.9%)	29 (26.6%)	5 (6.4%)	13 (28.9%)	4 (10.0%)	2 (7.4%)	1 (4.0%)
Dissociation	68 (28.7%)	7 (4.9%)	24 (22.2%)	1 (1.3%)	7 (15.6%)	1 (2.5%)	2 (7.4%)	0
Vertigo	62 (26.2%)	5 (3.5%)	16 (14.7%)	0	4 (8.9%)	2 (5.0%)	4 (14.8%)	0
Dysgeusia	46 (19.4%)	19 (13.2%)	19 (17.4%)	11 (14.1%)	2 (4.4%)	3 (7.5%)	2 (7.4%)	0
Somnolence	39 (16.5%)	15 (10.4%)	21 (19.3%)	5 (6.4%)	1 (2.2%)	3 (7.5%)	0	0
Paresthesia	31 (13.1%)	2 (1.4%)	12 (11.0%)	2 (2.6%)	2 (4.4%)	2 (5.0%)	2 (7.4%)	0
Anxiety	19 (8.0%)	10 (6.9%)	12 (11.0%)	2 (2.6%)	2 (4.4%)	2 (5.0%)	0	3 (12.0%)
Fatigue	19 (8.0%)	9 (6.3%)	6 (5.5%)	2 (2.6%)	7 (15.6%)	4 (10.0%)	2 (7.4%)	1 (4.0%)
BP increased	16 (6.8%)	3 (2.1%)	14 (12.8%)	2 (2.6%)	8 (17.8%)	3 (7.5%)	1 (3.7%)	0
Hypoesthesia	25 (10.5%)	1 (0.7%)	13 (11.9%)	2 (2.6%)	3 (6.7%)	1 (2.5%)	1 (3.7%)	0
Hypoesthesia oral	29 (12.2%)	2 (1.4%)	8 (7.3%)	1 (1.3%)	4 (8.9%)	0	1 (3.7%)	0
Vomiting	27 (11.4%)	3 (2.1%)	5 (4.6%)	1 (1.3%)	5 (11.1%)	0	0	1 (4.0%)
UTI	6 (2.5%)	2 (1.4%)	0	1 (1.3%)	6 (13.3%)	1 (2.5%)	0	0

The table lists, in descending order of frequency for all patients, all adverse events with an incidence ≥ 10% in any esketamine group

*AE* adverse event, *BP* blood pressure, *UTI* urinary tract infection

A serious adverse event was reported during treatment with esketamine for two (0.7%) women (single events of depression and anxiety disorder) and four (2.9%) men (single events of headache, hip fracture, increased blood pressure, and multiple injuries/road traffic accident [the latter event occurred on day 16 and was considered doubtfully related to esketamine or to antidepressant; patient subsequently died on day 55]).

A minority of patients discontinued intranasal study drug due to adverse events in the TRANSFORM studies (esketamine: 20/415, 4.8%; placebo: 5/287, 1.7%). Among esketamine-treated patients, 12 women discontinued study drug prematurely, due to multiple events in some cases (5 events of increased systolic blood pressure and 1 event of anxiety disorder for 1 older patient each in TRANSFORM-3; 3 events each of dizziness and nausea, 2 events each of depression, headache, and vomiting, and single events of anxiety, disturbance in attention, drug intolerance, extrasystoles, feeling drunk, motion sickness, tachycardia, and vertigo for younger patients in TRANSFORM-1/TRANSFORM-2) and 8 men discontinued study drug prematurely (single events of hip fracture and increased blood pressure for 1 older patient each in TRANSFORM-3; 2 events of panic attack and single events of anxiety, depressive symptoms, mania, and multiple injuries/road traffic accident for younger patients in TRANSFORM-1/TRANSFORM-2).

## Discussion

Similar and robust improvement of depressive symptoms from baseline was observed with esketamine nasal spray compared to placebo nasal spray, in conjunction with an oral antidepressant, among both women and men with TRD. While the between-group difference observed with esketamine/antidepressant vs. antidepressant/placebo was numerically higher among women than men in TRANSFORM-1/TRANSFORM-2 (LS means (SE) -4.5 (1.41) 95% CI -7.26, -1.70 for women vs. -1.6 (2.04) 95% CI -5.60, 2.41 for men) and vice versa in TRANSFORM-3 (LS means (SE) -3.4 (2.41) 95% CI -8.14, 1.41 for women vs. -5.0 (3.05) 95% CI -11.05, 1.03 for men), the treatment-by-sex interaction was not statistically significant (*p* > 0.35) and the 95% CIs for the differences overlap. Furthermore, the between-group difference observed vs. antidepressant/placebo for both sex subgroups in TRANSFORM-1/TRANSFORM-2 and TRANSFORM-3 was in the range considered clinically meaningful (2-point to 3-point difference) (Montgomery and Möller 2009; Kim et al. 2019) and is consistent with that observed in clinical trials of the most recently approved biogenic amine antidepressants compared with only a placebo rather than an active comparator (Preskorn 2013).

The proportions of patients who were responders at day 28 and the proportion of patients in remission at day 28 were numerically higher among both women and men treated with esketamine/antidepressant as compared to antidepressant/placebo. While it is noted that the remission rate, but not response rate, was numerically higher among the older women, compared to the older men in TRANSFORM-3, the small cohort sizes limit a conclusion being made from the comparison. Additionally, as noted in the “Materials and methods” section, the TRANSFORM-3 study, unlike TRANSFORM-1 and 2, included a lower 28 mg dose and post hoc analyses of TRANSFORM-3 data revealed several factors that potentially contributed to its failure to achieve statistical significance on the primary endpoint (Ochs-Ross et al. 2020).

The treatment benefit of esketamine, regardless of sex, was also observed based on the patient-reported outcomes of functioning (SDS total score), severity of anxiety (GAD-7 total score), and depressive symptoms (PHQ-9 total). The absence of evidence of sex-based differences may be explained, in part, by the absence of differences in the pharmacokinetics of esketamine between male and female subjects (Spravato^TM^ Prescribing Information 2020).

Sex differences in efficacy outcomes have been reported for other antidepressants, although findings have been inconsistent (Sramek et al. 2016). In some studies, older men responded better to tricyclic antidepressants than women (Frank et al. 1988; Jacoby et al. 1993; Kornstein et al. 2000), and in other studies women responded better to SSRIs, and to a lesser extent SNRIs, than men (Khan et al. 2005; Kornstein et al. 2000; Berlanga and Flores-Ramos 2006; Young et al. 2009; Yang et al. 2011). Alternatively, other studies have reported no sex-based differences in antidepressant efficacy (Kornstein et al. 2006, 2010, 2018; Cuijpers et al. 2014). Differences in study design (prospective, retrospective, meta-analysis), patient selection criteria (e.g., age of patients, clinical presentation, severity/duration of depression), study drug (mechanism of action, dosage, duration of treatment), and response criteria may explain the inconsistency in these findings across studies.

As noted in the “Introduction” section, findings regarding the effect of menopausal status on response to antidepressants have also been mixed (Kornstein et al. 2000; Quitkin et al. 2002; Grigoriadis et al. 2003; Cassano et al. 2005; Thase et al. 2005; Pinto-Meza et al. 2006; Kornstein et al. 2014), with some research groups reporting greater response among women treated with an SSRI vs. a tricyclic antidepressant, driven by between-group differences in pre-menopausal women, and not in those who were post-menopausal (Kornstein et al. 2000). In a pooled analysis of data from 8 randomized, controlled trials, older women exhibited lower remission rates on SSRI than younger women, a trend that was reversed for those taking hormone replacement therapy; remission rates were higher for women treated with SNRI, irrespective of age and hormone replacement therapy (Thase et al. 2005). Although small sample size precluded analysis of efficacy by concomitant oral antidepressant class (SSRI, SNRI), by sex or menopausal status, oral antidepressant class was included in the MMRM as a fixed effect.

With esketamine, while the response rate with esketamine/antidepressant was numerically lower in the small sample of peri-menopausal women (*n* = 24), it appears that post-menopausal women with TRD achieve the same benefit that pre-menopausal women do. These findings are consistent with those of Freeman et al. (2019), who found no difference between women and men treated with intravenous ketamine for rapid reduction of depressive symptoms, with no difference observed based on menopause status, suggesting antidepressants with a glutamatergic mechanism of action, unlike biogenic amines, may not be impacted by the reproductive life cycle of women.

In the TRANSFORM-1/2 studies, use of hormonal therapy increased response rate at day 28 in the antidepressant/placebo arm, but not in the esketamine/antidepressant arm. Others have also observed that hormone therapy impacts response among women receiving SSRI or SNRI (Schneider et al 2001; Thase et al. 2005).

The most common adverse events experienced by esketamine-treated patients were dissociation, headache, nausea (each reported at a rate higher in women than men), dizziness (reported at a rate higher in older women than older men [in TRANSFORM-3]), and vertigo (reported at a rate higher in younger women than younger men [in TRANSFORM-1/TRANSFORM-2]). Increased blood pressure was reported most often among older women. This trend is in line with the higher risk for women having adverse drug reactions, in general (Anderson 2008), and for specific types of events during treatment with antidepressants (e.g., weight gain with SSRIs) (Noordam et al. 2015).

### Limitations

The strengths of this post hoc analysis include the relatively large numbers of participants, the active-controlled design, validated diagnostic assessments for comorbid psychiatric disorders, and the systematic ascertainment of menopausal status and other sex-specific data.

Our findings are limited by the fact that patients were not stratified into the TRANSFORM studies based on sex, although the higher participation by women (66.1% overall) was similar across the TRANSFORM studies and is consistent with the well-known sex disparity in the prevalence of major depression worldwide (Seedat et al. 2009). We note that the results of analyses by menopausal status must be interpreted with caution given small sample sizes. Similarly, sub-analyses from TRANSFORM-3 (patients 65 years and older) should also be interpreted with caution due to the small sample size and differences in dosing regimen (including a lower 28 mg dose, in addition to 56 mg and 84 mg). Other limitations to the generalizability of findings from the current post hoc analysis include exclusion of patients with some common psychiatric and medical comorbidities and low participation by non-white patients.

## Conclusion

These analyses support antidepressant efficacy and safety of esketamine nasal spray for women with TRD, without notable differential effects based on sex. These findings add to the existing literature and support data-informed decision-making for women with TRD.

## Data Availability

The data sharing policy of Janssen Pharmaceutical Companies of Johnson & Johnson is available at https://www.janssen.com/clinical-trials/transparency. As noted on this site, requests for access to the study data can be submitted through Yale Open Data Access (YODA) Project site at http://yoda.yale.edu.
